# Supplementary material for: Secondary Metabolites of The Endophytic Fungus Alternaria alternata JS0515 Isolated from Vitex rotundifolia and Their Effects on Pyruvate Dehydrogenase Activity
Source: Molecules. 2019 Dec 4;24(24):4450. doi: 10.3390/molecules24244450 (PMC6943735; doi:10.3390/molecules24244450)

Supporting Information

Secondary Metabolites of The Endophytic Fungus *Alternaria alternata* JS0515 Isolated from *Vitex rotundifolia* and Their Effects on Pyruvate Dehydrogenase activity

Changyeol Lee,[1,#] Wei Li,[2,#] Sunghee Bang,[1] Sun Joo Lee,[3] Nam-young Kang,[4] Soonok Kim,[5] Tae In Kim,[2] Younghoon Go,[2,*] and Sang Hee Shim[1,*]

[1] College of Pharmacy, Duksung Women's University,144 Gil 33,Dobong-gu, Seoul 01369, Republic of Korea; jaber29@naver.com (C.Y.L.); scbsh4331@hanmail.net (S.H.B.); sangheeshim@duksung.ac.kr (S.H.S.)

[2] Korean medicine (KM)-Application Center, Korea Institute of Oriental Medicine (KIOM), Daegu 41062, Republic of Korea; liwei1986@kiom.re.kr (W.L.); tikim@kiom.re.kr (T.I.K.); gotra827@kiom.re.kr (Y.H.G.)

[3] New Drug Development Center, Daegu-Gyeongbuk Medical Innovation Foundation, 80 Cheombok-ro, Dong-gu, Daegu 41061, Republic of Korea; disjrk@dgmif.re.kr (S.J.L.)

[4] Department of Creative IT Engineering, Pohang University of Science and Technology (POSTECH), 77 Cheongam-ro, Namgu, C5 building, room203, Pohang, Kyungbuk 37673, Republic of Korea; knysg@postech.ac.kr (N.Y.K.)

[5] Biological Resources Assessment Division, National Institute of Biological Resources, Incheon 22689, Republic of Korea; sokim90@korea.kr (S.O.K.)

[#] These authors contributed equally to this work.

* Correspondence: gotra827@kiom.re.kr (Y.H.G.); sangheeshim@duksung.ac.kr (S.H.S.)

Table of Contents

Figure S1. ^{1}H NMR spectrum (500 MHz) of **1** in DMSO-d_6.

Figure S2. ^{13}C NMR spectrum (125 MHz) of **1** in DMSO-d_6.

Figure S3. HSQC spectrum (500 MHz) of **1** in DMSO-d_6.

Figure S4. HMBC spectrum (500 MHz) of **1** in DMSO-d_6.

Figure S5. ROESY spectrum (500 MHz) of **1** in DMSO-d_6.

Figure S6. HR-ESI-MS of **1**.

Figure S7. ECD spectrum of **1**.

Figure S8. (A) and (C) Immunofluorescence analysis to quantify p-PDH E1α (Ser300) in AD-293 cells cultured with **3** and **11**. Phosphorylation inhibition by **3** and **11** was normalized against DCA inhibition. (B) and (D) Dose-response curves for p-PDH E1α (Ser300) from (A) and (C). The dose-response curves were generated using the Prism 6 software package.

Figure S1. ^{1}H NMR spectrum (500 MHz) of **1** in DMSO-d_6.

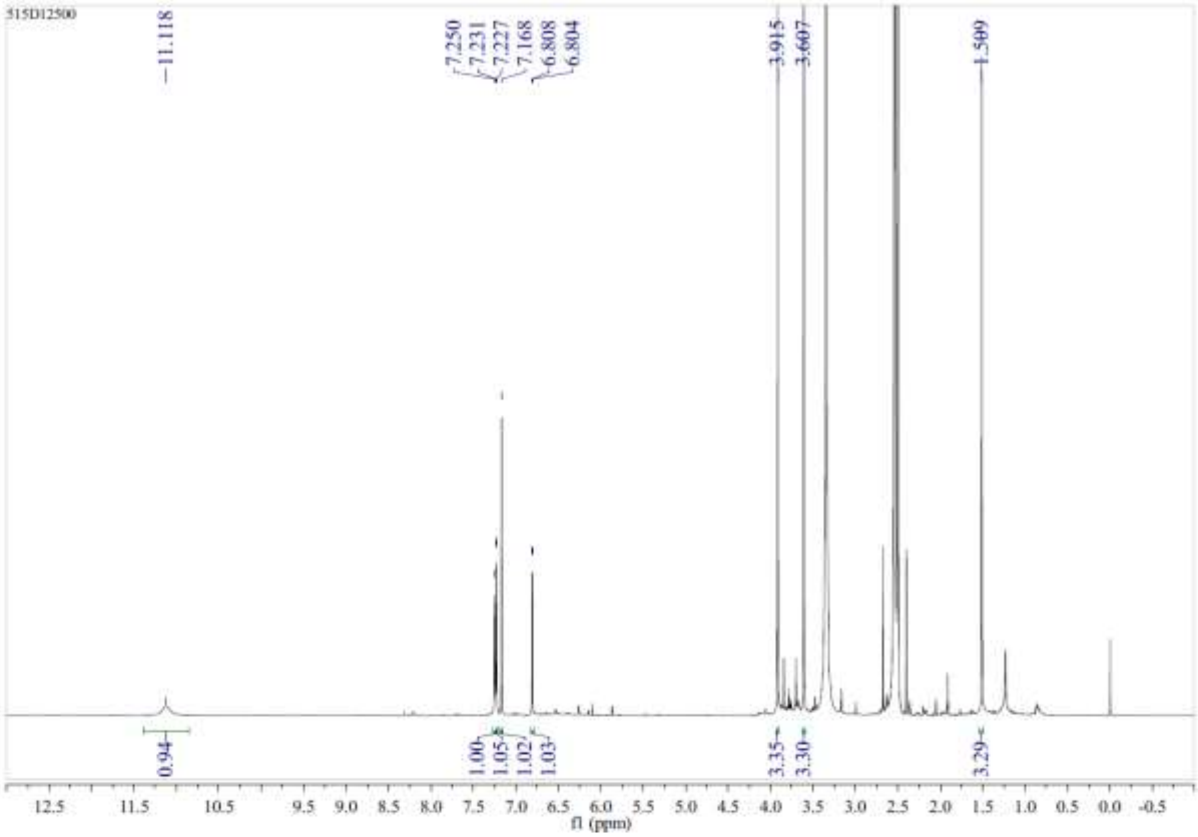

Figure S2. ^{13}C NMR spectrum (125 MHz) of **1** in DMSO-d_6.

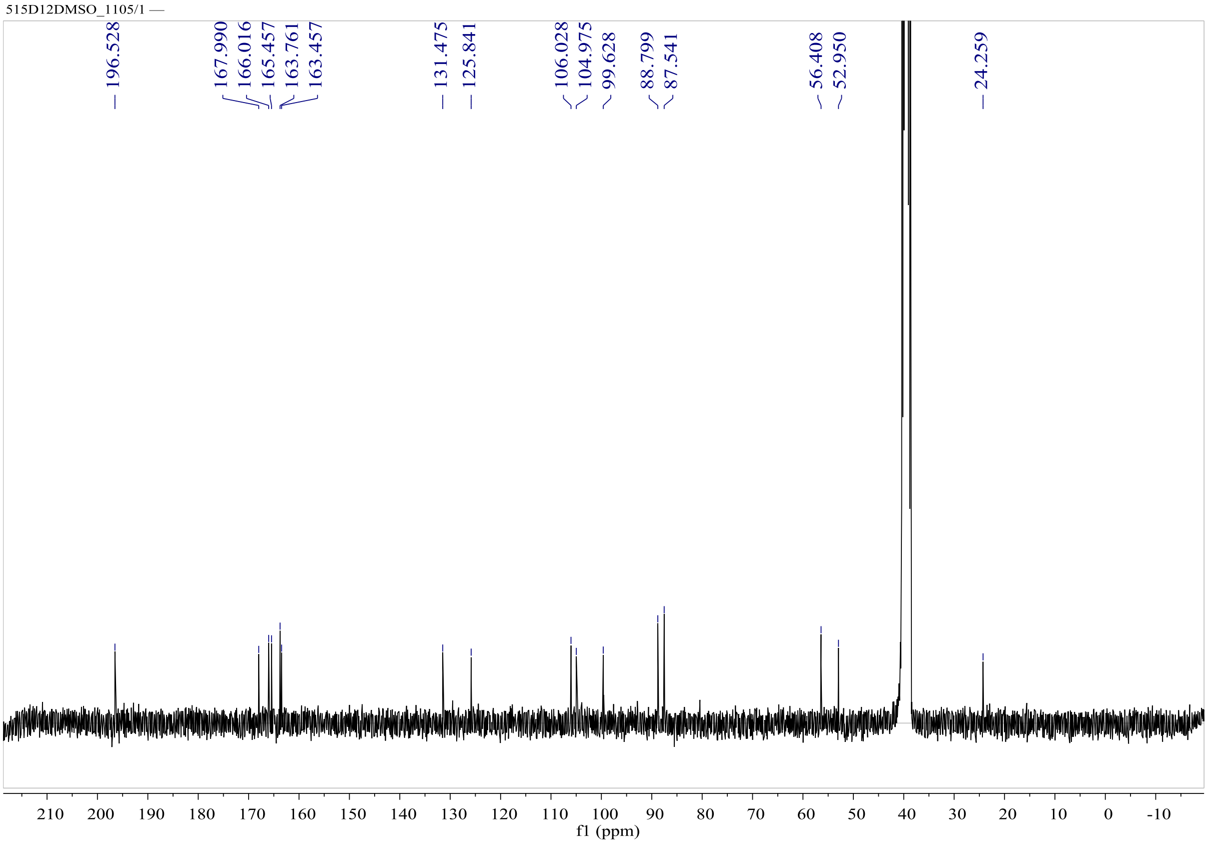

Figure S3. HSQC spectrum (500 MHz) of **1** in DMSO-d_6.

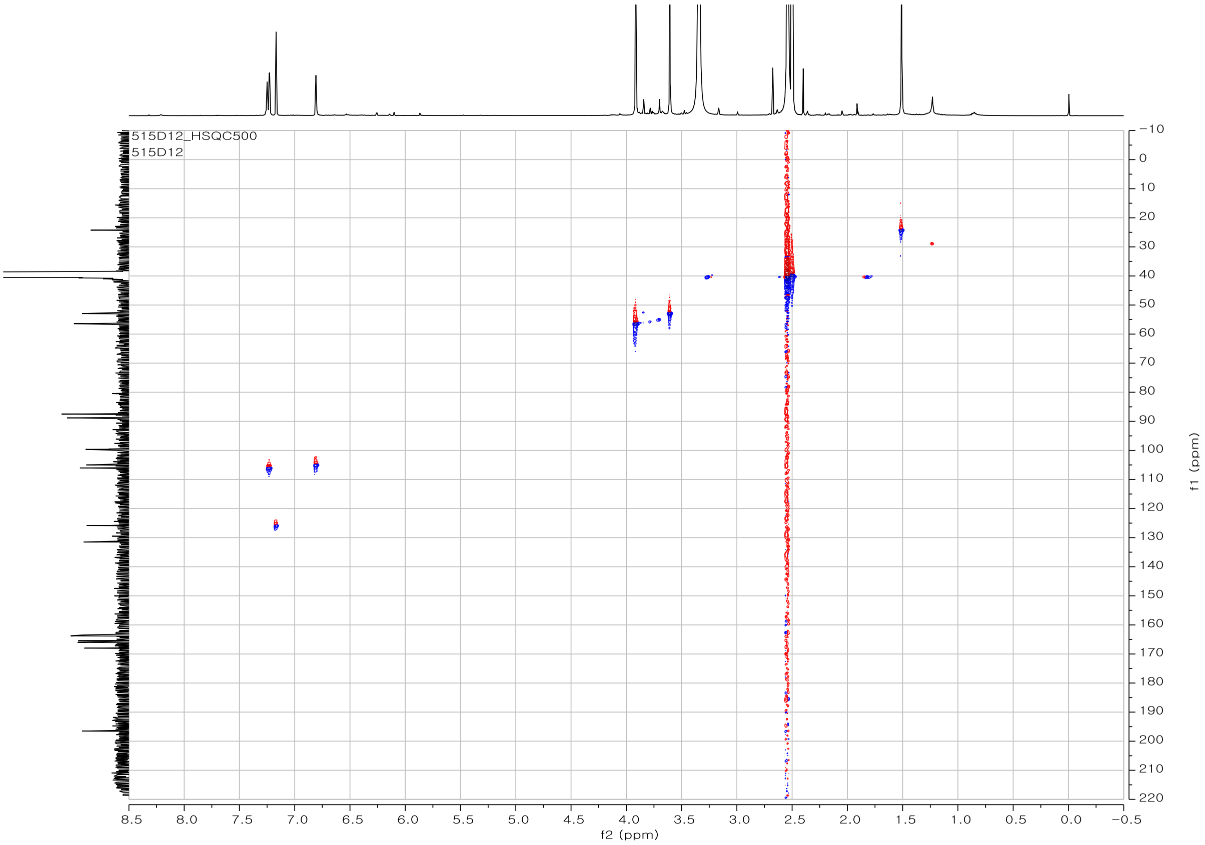

Figure S4. HMBC spectrum (500 MHz) of **1** in DMSO-d_6.

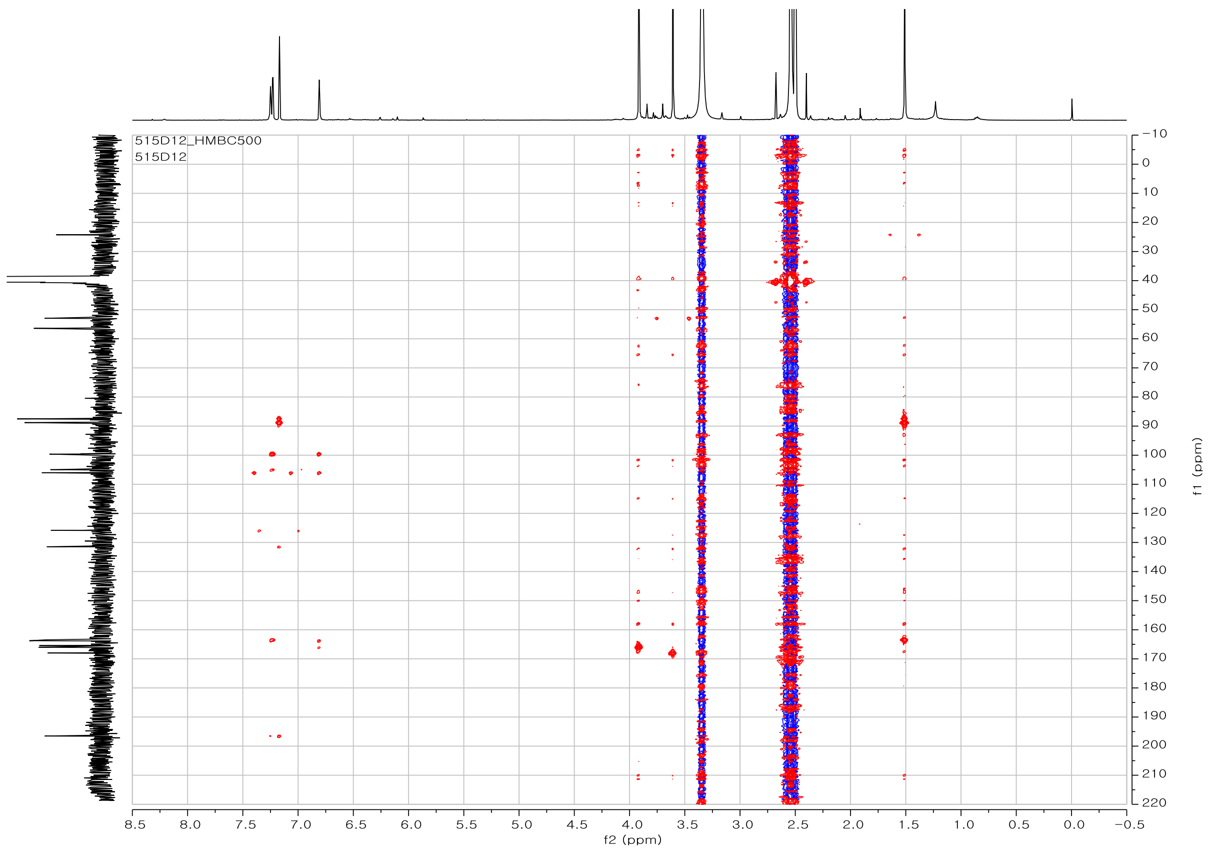

Figure S5. ROESY spectrum (500 MHz) of **1** in DMSO-d_6.

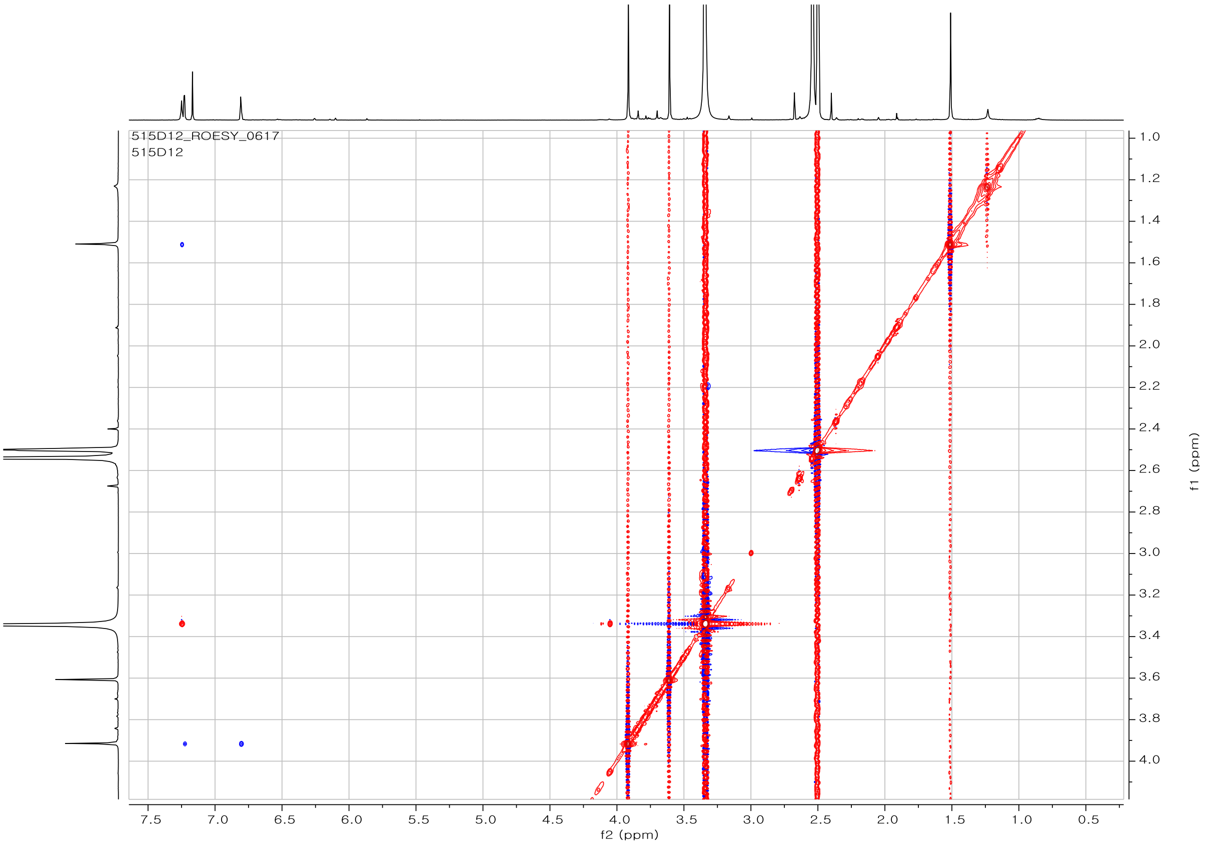

Figure S6. HR-ESI-MS of **1**.

Compound Spectrum SmartFormula Report

Analysis Info

Analysis Name	D:\Data\LHK2016\DSWU\SSH\BSH006.d	Acquisition Date	11/24/2016 3:56:12 PM
Method	Tune_pos_Standard_90_800_161110d.m	Operator	LHK
Sample Name	515D12	Instrument	compact 8255754.10024
Comment			

Acquisition Parameter

Source Type	ESI	Ion Polarity	Positive	Set Nebulizer	0.4 Bar
Focus	Active	Set Capillary	4000 V	Set Dry Heater	200 °C
Scan Begin	90 m/z	Set End Plate Offset	-500 V	Set Dry Gas	4.0 l/min
Scan End	900 m/z	Set Charging Voltage	2000 V	Set Divert Valve	Source
		Set Corona	0 nA	Set APCI Heater	0 °C

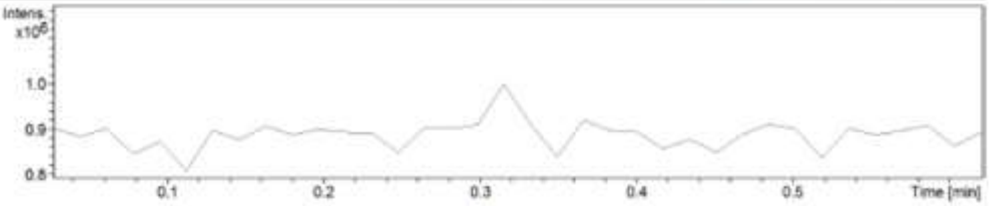

#	RT [min]	Area	Int. Type	I	S/N	Chromatogram	Max. m/z	FWHM [min]
n.a.	0.3	n.a.	Average spectrum	n.a.	n.a.	n.a.	357.0591	n.a.

+MS, 0.2-0.5min #10-28

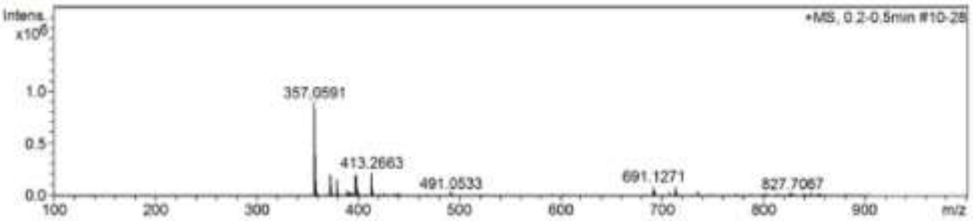

Meas. m/z	#	Ion Formula	m/z	err [ppm]	mSigma	# Sigma	Score	rdb	e⁻ Conf	N-Rule
357.0591	1	C14H9N6O6	357.0578	3.5	2.7	1	69.48	13.5	even	ok
	2	C11HN16	357.0565	7.3	9.1	2	22.58	19.5	even	ok
	3	C13H13N2O10	357.0565	7.3	9.9	3	29.91	8.5	even	ok
	4	C10H5N12O4	357.0551	-11.1	12.5	4	5.33	14.5	even	ok
	5	C18H13O8	357.0605	-4.0	13.4	5	69.31	12.5	even	ok
	6	C15H5N10O2	357.0591	-0.2	18.1	6	100.00	18.5	even	ok
	7	C19H9N4O4	357.0618	-7.7	28.7	7	13.11	17.5	even	ok
	1	C16H14NaO8	357.0581	-2.8	2.5	1	100.00	9.5	even	ok
	2	C13H6N10NaO2	357.0567	6.5	7.0	2	27.11	15.5	even	ok
	3	C12H10N6NaO6	357.0554	-10.3	11.0	3	6.89	10.5	even	ok
	4	C17H10N4NaO4	357.0594	1.0	16.3	4	80.34	14.5	even	ok
	5	C18H6N8Na	357.0608	-4.7	30.0	5	27.43	19.5	even	ok

Fig. S7. ECD spectrum of **1**.

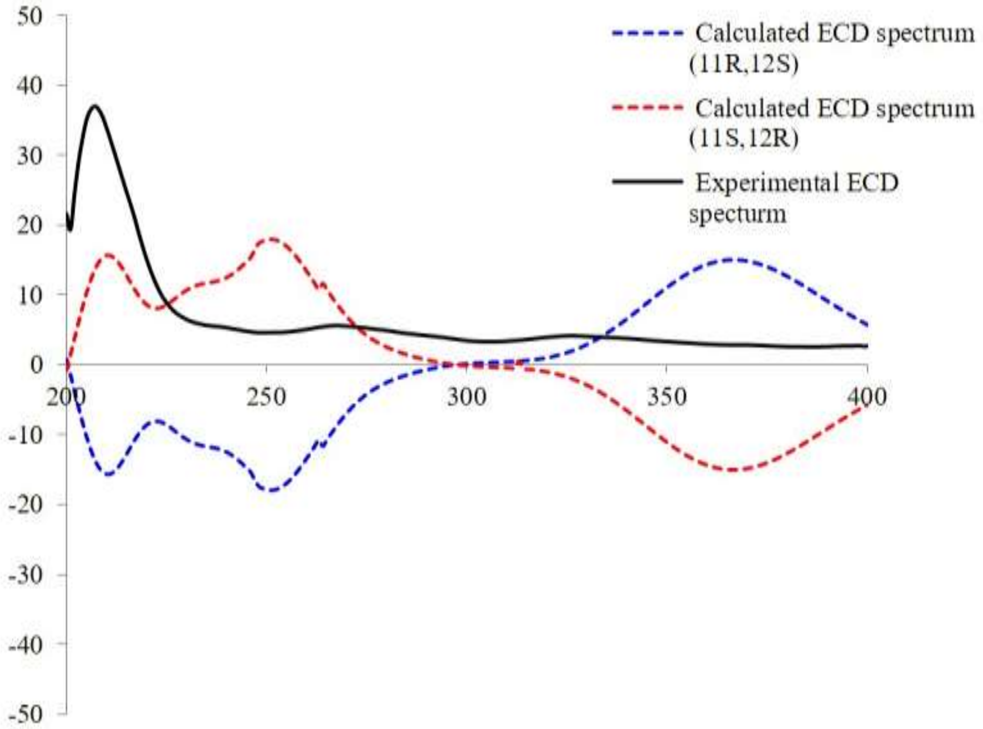

Fig. S8. (A) and (C) Immunofluorescence analysis to quantify p-PDH E1α (Ser300) in AD-293 cells cultured with **3** and **11**. Phosphorylation inhibition by **3** and **11** was normalized against DCA inhibition. (B) and (D) Dose-response curves for p-PDH E1α (Ser300) from (A) and (C). The dose-response curves were generated using the Prism 6 software package.

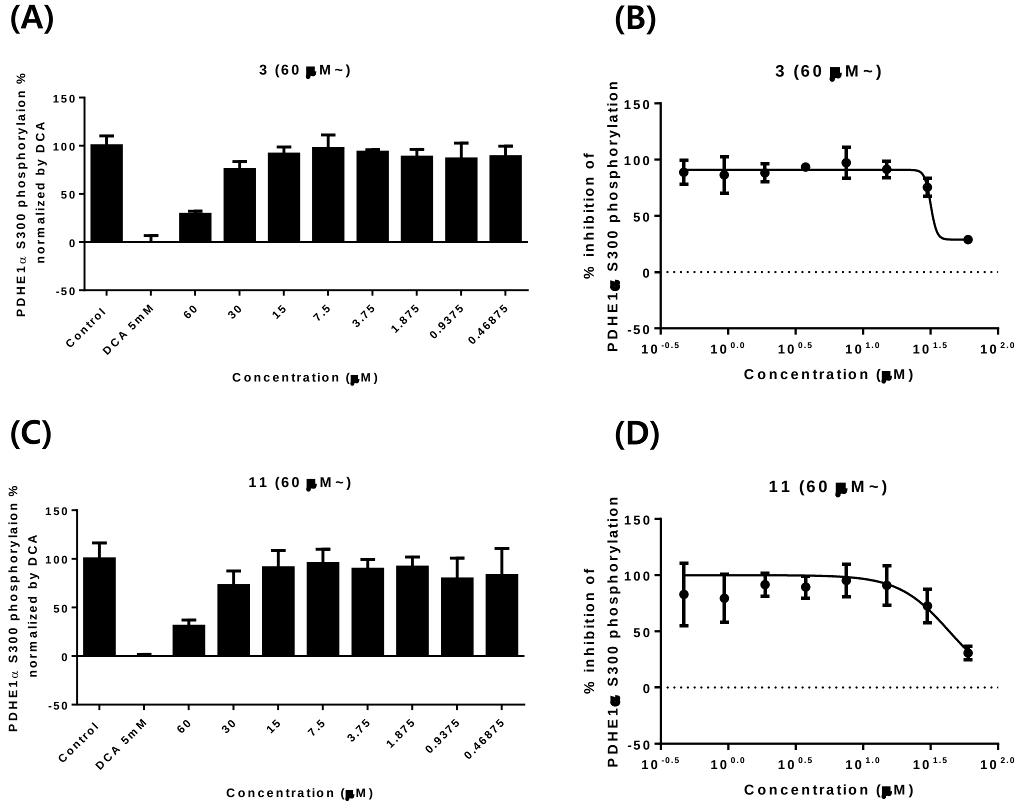

Supplement: Supplementary file 1 [file molecules-24-04450-s001.pdf]
